# βig-h3 enhances chondrogenesis via promoting mesenchymal condensation in rat Achilles tendon heterotopic ossification model

**DOI:** 10.18632/aging.103060

**Published:** 2020-04-20

**Authors:** Qiang Zhang, Yan Zhang, Meijun Yan, Kai Zhu, Qihang Su, Jie Pan, Mingjie Yang, Dong Zhou, Jun Tan

**Affiliations:** 1 Department of Orthopaedic Surgery, Shanghai East Hospital, Tongji University School of Medicine, Shanghai, China; 2 Department of Orthopaedic Surgery, The Affiliated Changzhou No. 2 People’s Hospital with Nanjing Medical University, Changzhou, China; 3 Department of Orthopedics, Pinghu Second People’s Hospital, Pinghu, China

**Keywords:** βig-h3, chondrogenic differentiation, heterotopic ossification, mesenchymal condensation

## Abstract

Heterotopic ossification (HO) is a poorly characterized disease with ectopic bone formation in the musculoskeletal soft tissues. HO is widely considered as a tissue repair process goes away, with endochondral ossification to be the major pathological basis. The molecular mechanism of how the resident/recruited progenitor cells for tissue regeneration error differentiated into the chondrocytes remains unknown. Here, we found Transforming Growth Factor B Induced Gene Human Clone 3 (βig-h3) was highly expressed in the inflammation and chondrogenesis stages of a heterotopic ossification model after rat Achilles tendon injury, as well as upon chondrogenic differentiation conditions in vitro. βig-h3 functioned as an extracellular matrix protein, which was induced by TGFβ signaling, could bind to the injured tendon-derived stem cells (iTDSCs) and inhibit the attachment of iTDSCs to collagen I. Exogenous βig-h3 was also found able to accelerate the process of mesenchymal condensation of cultured iTDSCs and promote chondrogenic differentiation in vitro, and additional injection of iTDSCs could promote endochondral ossification in Achilles tendon injury model. Taken together, βig-h3 might function as an adhesion protein that inhibited the attachment of iTDSCs to collagen I (the injury site) but promoted the attachment of iTDSCs to each other, which resulted in promoting chondrogenic differentiation.

## INTRODUCTION

Heterotopic ossification (HO) in the tendon is common in the clinic, with no effective treatment has been developed ever. The disease is poorly characterized, which is widely considered as a tissue repair process gone away. The pathology basis of HO in the tendon is endochondral ossification that consists of four stages: inflammation, multi-potential progenitors recruitment, chondrogenesis, and osteogenesis [1, 2]. The chondrogenesis process seems most important in HO development, however, the molecular mechanism of how the recruited progenitor cells differentiate into the chondrocytes but not the tenocytes, remains unknown.

TGF-β has been widely proofed to be involved in various types of HO. In fibrodysplasia ossificans progressive (FOP), a special type of genetic HO, TGF-β has been shown to play an important role that the pharmacologic inhibition of TGF-β signaling decreases osteogenic differentiation of FOP fibroblasts [3]. Similarly, in Achilles tendon ossification model, the inhibition of TGF-β activity successfully mitigates HO at different stages of HO [4]. Previous studies also showed that TGF-β is activated after injuries and is required in all phases of chondrogenesis, from mesenchymal condensation to finally terminal differentiation [5, 6]. It’s interesting that based on different cell types, different experiment environments, or even different time points, the TGF-β has been shown to have both the abilities of chondrogenesis and tenogenesis. Thus, considering the importance of TGF-β in regulating the balance between sox9 and scleraxis expression and therefore the shift between chondrogenesis and tenogenesis [7, 8], the role and mechanism of it in tendon ossification has gain more and more attention.

βig-h3 (Transforming Growth Factor B Induced Gene Human Clone 3), also known as TGFBI, is an ECM molecule induced by TGFβ signaling [9]. βig-h3 is generally known as a cell adhesion-class protein that comprises of a secretory signal sequence, an N-terminal cysteine-rich EMI domain, four fasciclin 1 domains, and an RGD (Arg–Gly–Asp) motif [10]. βig-h3 has been associated with the differentiation of various types of connective tissues during development, including tendons, cartilage, entheses, and joint capsules [11–14]. Similar with the TGFβ, βig-h3 has also been associated with both chondrogenesis and tenogenesis. Lorda-Diez et al demonstrated that βig-h3 promoted the fibrogenic influence of TGFβ signaling, neutralizing the prochondrogenic influence of hypoxic microenvironment of limb mesenchymal aggregates [14]. Transcripts of βig-h3 are very abundant in tendon primordia, and are maintained in the developing tendons and joint fibrous capsules for longer periods of development [15]. At the same time, βig-h3 also plays a critical role as a regulator of chondrogenic differentiation. During the chick embryogenesis, βig-h3 was localized at the pre-cartilage condensation of limb buds and highly expressed in the pre-hypertrophic in the vertebrae [11]. During mouse development, βig-h3 expression was high in pre-chondrocytic mesenchymal cells, and continuously observed during the cartilaginous formation [13]. Lee et al reported βig-h3 plays an important role in maintaining the cartilage matrix and skeletal tissues in mice [16]. A previous study also demonstrated βig-h3 was mainly induced by TGF-β1 at the pre-hypertrophic chondrocytes and may mediate the function of TGF-β during endochondral ossification [17]. It seems βig-h3 exhibits either tenogenesis or chondrogenesis abilities based on different cell types, environments, or time points. The precise expression pattern and function of βig-h3 in chondrocyte differentiation during endochondral ossification remains obscure. Here, we aim to investigate the expression patterns of βig-h3 in the heterotopic ossification model of the Achilles tendon, as well as the role of it in chondrogenic differentiation.

## RESULTS

### TGF-β activity is elevated in rat Achilles tendon HO model

**Figure 1:**
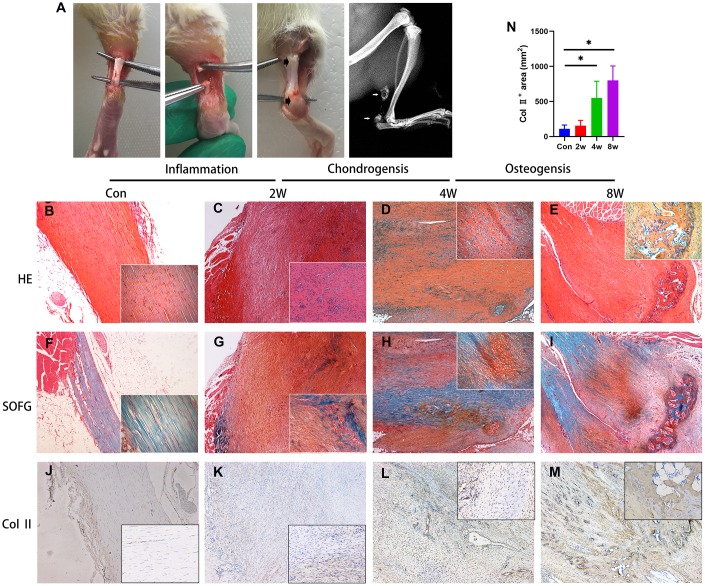
**Endochondral ossification is the pathological basis of the heterotopic ossification model in the rat after tendon injury.** (**A**) The HO model was made by complete transverse incision at the midpoint of Achilles tendon without any attempt of repair. Bone-like tissues formed near the position of the tendon-to-bone junction and tendon-to-muscle junction (arrow) at 8 weeks post-surgery. (**B**–**I**) The H&E and Safranin O/fast green staining. Inflammation infiltration was obvious at 2w, lots of chondrocytes could be found at 4w, and bone tissues formed at 8w. (**J–N)** The immunohistological staining of Col II ^+^ cells (brown). *p < 0.05 as determined by one-way ANOVA test.

To confirm the pathogenesis of HO, the rats were anesthetized 8 weeks post-surgery and subjected to radiological analysis. The X-ray images demonstrated the formation of bone-like tissues near the areas of the tendon-to-bone junction and tendon-to-muscle junction (Figure 1A). H&E (Figure 1B–1E) and Safranin O and fast green (SOFG) (Figure 1F–1I) staining also confirmed the process of endochondral ossification occurred in injured Achilles tendon, as well as shown in immunohistology staining of Col 2 (Figure 1J–1N).

**Figure 2:**
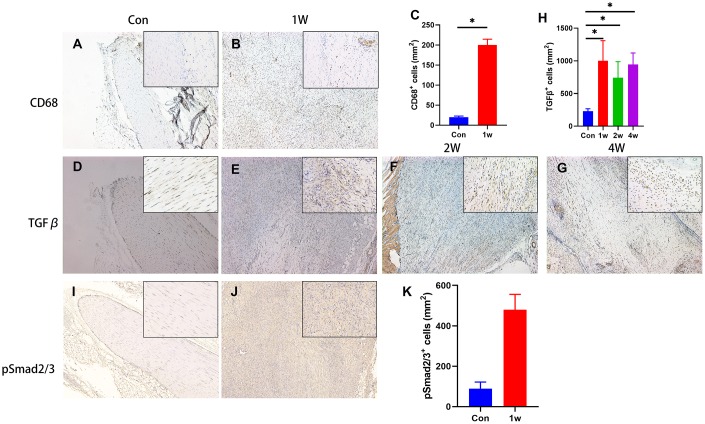
**TGF-β signaling is elevated during the chondrogenic differentiation stage during HO development.** (**A**–**C**) The immunohistological staining of CD68^+^ cells (brown). (**D**–**H**) The immunohistological staining of TGF-β1^+^ cells (brown). (**I**–**K**) The immunohistological staining of phosphorylated Smad2/3^+^ cells (brown). *p < 0.05 as determined by one-way ANOVA test.

Previous studies have demonstrated the elevated TGF-β released by macrophages at the inflammation stage triggers HO [4]. Here, we found the TGF-β signal not only participated in the inflammation stage but also triggers chondrogenic differentiation during HO development. We found that accumulation of CD68^+^ immune cells (macrophage) and high levels of active TGF-β 1 week after injury in rat Achilles tendon HO model (Figure 2A–2E, 2H), suggesting that active TGF-β and immune cells are closely related to the onset of HO. Moreover, the elevated level of TGF-β1 was also found at 2- and 4-weeks post-surgery (Figure 2F–2H), as known as the chondrogenic differentiation period. The number of phosphorylated Smad2/3-positive (pSmad2/3^+^) cells, the major TGF-β downstream signaling transducer, was also found significantly elevated (Figure 2I–2K). Altogether, our results reveal that the acquired HO model in rat Achilles tendon developed via endochondral ossification, with high levels of active TGF-β in the microenvironment during inflammation and chondrogenic stages.

### βig-h3 is involved in chondrogenic differentiation during HO formation

**Figure 3:**
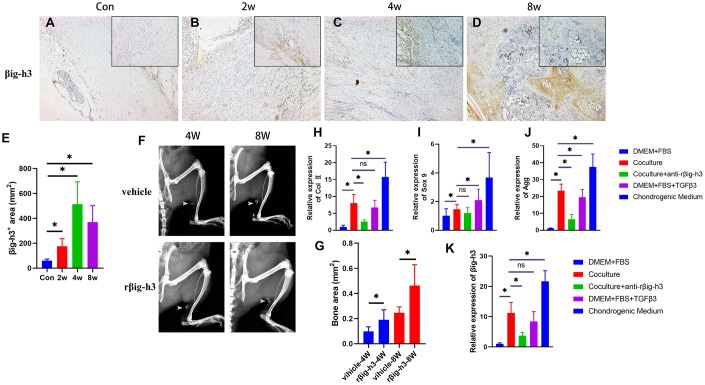
**βig-h3 is involved in chondrogenic differentiation for HO formation.** (**A**–**E**) The immunohistological staining of βig-h3^+^ cells (brown). (**F, G**) The represent X ray and statistics of rat with/without rβig-h3 injection. (**H**–**J**) The expression of Sox 9, Aggrecan, and Col II with/without coculture of macrophage cells. (**K**) The expression of βig-h3 with/without coculture of macrophage cells. *p < 0.05 as determined by one-way ANOVA test.

To understand how increased TGF-β/Smad2/3 signaling participates in ectopic bone formation, or in another word, how TGF-β/Smad2/3 signaling regulates the chondrogenic differentiation, we first analyzed the expression pattern of βig-h3. βig-h3 was found expressed during inflammation stage, with a rapid elevation at chondrogenic differentiation stages and decreased during late chondrogenesis stage and osteogenic differentiation stage (Figure 3A–3E), which is similar to a previous paper that demonstrated βig-h3 was involved in the early stages of chondrogenic differentiation in ATDC5 [17]. It should be noted that the positive staining area of βig-h3 in the sections of 8w seemed large, different from the previous conclusion that βig-h3 was only involved in the early stages of chondrogenic differentiation. But we could not ignore that there were few positive areas in the center of the ossification and most positive areas (the arrow) located in the peripheral places, namely, the immature chondrocytes. Also, the additional injection of rβig-h3 could induce significant earlier and more ossification in the rat Achilles tendon injury model (Figure 3F–3G).

To explore the effect of βig-h3 in chondrogenic differentiation in vitro, we first cocultured the iTDSCs with macrophage cells. After 3 days’ coculture, the iTDSCs were collected for RT-PCR analysis of chondrogenesis genes. The co-cultured iTDSCs demonstrated markedly more Sox 9, Aggrecan, and Col II expression than the cells without (Figure 3H–3J), similar to the cells cultured with additional TGFβ3, though much lower than the cells cultured in complete chondrogenic differentiation medium (Figure 3H–3J). Moreover, in presence with the antibody-neutralized rβig-h3, the expression of chondrogenic markers decreased significantly (Figure 3H–3J). Similarly, upon coculture, exogenous TGFβ3, or chondrogenic medium, the expression of βig-h3 is obviously elevated and can be inhibited by the additional anti-rβig-h3 (Figure 3K).

The chondrogenesis process in vivo contains a series of phases including mesenchymal recruitment, mesenchymal condensation, chondrocyte proliferation, extracellular matrix deposition and finally terminal differentiation. Considering the expression pattern of βig-h3 in HO (elevated expression in inflammation stage and highest expression during chondrogenesis), it seems the role of it focuses on the recruitment of mesenchymal cells and triggering the chondrogenic differentiation. βig-h3 is a well-known secretory protein induced by TGF-β that plays a role in cell adhesion, differentiation, and apoptosis [18], among which the adhesion function is considered as the most important. It contributes to cell adhesion through interactions with integrins as well as several extracellular matrix (ECM) proteins including collagen, fibronectin, and laminin [19, 20]. So, regarding the expression pattern and function of βig-h3 in the HO model, we hypothesis if βig-h3 serves as a secretory protein that contributes to the adhesion of iTDSCs to the injury site and contributes to the mesenchymal condensation and chondrogenesis of iTDSCs.

### Cell attachment of βig-h3 to iTDSCs

**Figure 4:**
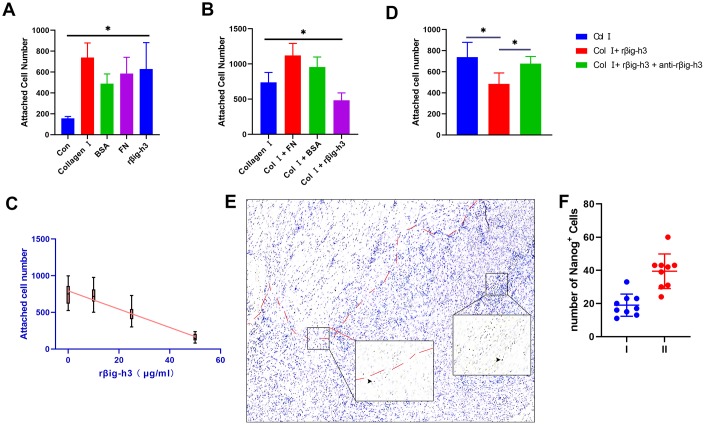
**Cell attachment of βig-h3 to iTDSCs.** (**A**) The cell attachment of iTDSCs to collagen I, BSA, FN, rβig-h3, or nothing. (**B**) iTDSCs attachment to collagen type I in the presence of BSA, FN, or rβig-h3. (**C**) The association between the dose of rβig-h3 and the number of attached cells. (**D**) The cell attachment of iTDSCs to collagen I in the presence of rβig-h3 with/without anti-rβig-h3. (**E**, **F**) The immunohistological staining of Nanog^+^ cells (brown, black arrow). The area above the red dotted line is the injured tendon. The positive cells were counted as the follows: I. area around the tendon (half of the 40X view is the tendon tissue) II. area except the tendon (without any tendon tissue)*p < 0.05 as determined by one-way ANOVA test.

To assess the effects of βig-h3 on iTDSCs’ adhesion to the injury site (mainly to tenocytes and type I collagen), iTDSCs were plated on 96-well plates coated with collagen I, BSA, FN, rβig-h3, or nothing. Significant more iTDSCs were attached to the rβig-h3 coated plates, in comparison with plates coated with nothing. However, no such differences were found in comparison with plates coated with collagen I, BSA, or FN (Figure 4A). The cells were also plated on collagen I coated 96-well plates supplied with BSA, FN, rβig-h3, or rβig-h3 + anti-βig-h3 antibody. rβig-h3 significantly inhibited the attachment of iTDSCs to collagen I, in comparison with BSA and FN (Figure 4B). The inhibition effect of rβig-h3 was also in a dose-dependent manner (Figure 4C), but can be interrupted by the additional antibody-neutralized rβig-h3 (25 μg/ml rβig-h3 + 50 μg/ml anti-βig-h3) (Figure 4D). Additionally, to figure out whether the reduction of attachment of iTDSCs to collagen I may inhibit the iTDSCs homing to the injured tendon sites, we further utilized the Nanog antibody as a iTDSCs marker to locate the cells. The recruited Nanog+ cells were mainly located far away from the tendon tissue but not around the injured tendon (Figure 4E, 4F). In summary, the cell attachment test demonstrated βig-h3 functioned as an adhesion substratum for iTDSCs in vitro but inhibited the adhesion of iTDSCs to collagen I, which means the recruited iTDSCs couldn’t bind to the injured tendon successfully. It may be the reason why in the tendon injury model, the recruited TDSCs formed cartilage but not the regeneration of tendon tissues.

βig-h3 was proved here to be able to bind to the surface of iTDSCs, similar to several studies that reported βig-h3 can bind to the surface of cells in connective tissue rich matrices to modulate their adhesive properties via cell-specific integrins [21, 22]. The Arg-Gly-Asp (RGD) sequence present on the C-terminal region of βig-h3 is thought to act as a universal ligand recognition site for these integrins, as well as the fasciclin-like (FAS) domains [23]. The βig-h3 protein contains 11 cysteine residues mainly clustered in the NH2-terminal region (EMI domain), four highly conserved FAS domains, and a COOH-terminal RGD sequence [24, 25]. Due to the presence of the multiple FAS and RGD domains, the βig-h3 protein may interact with multiple cells at the same time. Together with the potential role of it in inducing chondrogenic differentiation, we hypothesize if βig-h3 functions as an adhesion protein that promotes the process of mesenchymal condensation in chondrogenic differentiation.

### The role of βig-h3 in chondrogenic differentiation of iTDSCs

**Figure 5:**
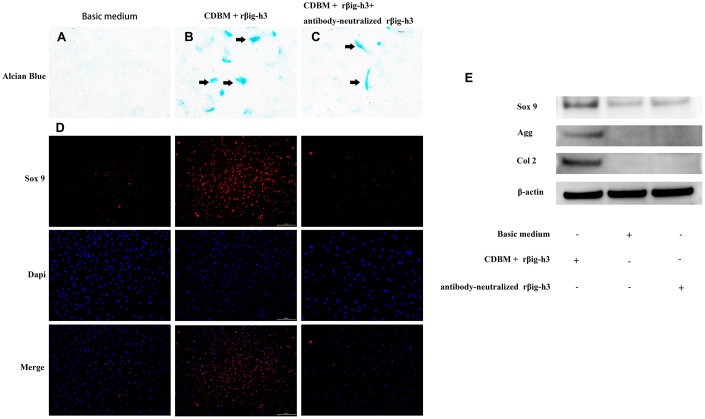
**βig-h3 promotes the process of mesenchymal condensation and chondrogenic differentiation of cultured iTDSCs.** (**A**–**C**) The mesenchymal condensation (black arrow) were detectable by day 14 with the additional rβig-h3, and the effect could be inhibited by the additional antibody-neutralized rβig-h3. (**D**) The immunofluorescence staining of Sox 9 showed more sox 9 expression by the additional rβig-h3. (**E**) The expression patterns of chondrogenesis genes Sox 9, Agg, and Col 2 in iTDSCs cultured with Chondrogenic Differentiation Basal Medium (CDBM), CDBM with additional rβig-h3, and CDBM supplied with additional rβig-h3 and antibody-neutralized rβig-h3. by western blotting.

To gain further insights into the potential role of βig-h3 in mesenchymal condensation and chondrogenic differentiation, we performed micromass and monolayer cultures with iTDSCs in chondrogenic differentiation medium with or without the supplement of additional rβig-h3. The iTDSCs supplied with rβig-h3 formed condensations identifiable after 7 days of culture (Figure 5A), which is much earlier than the cells without the supply of rβig-h3 (Figure 5B). And the effect could be inhibited by the additional antibody-neutralized rβig-h3 (Figure 5C). Further, to figure out whether βig-h3 was directly associate with cartilage differentiation besides cell aggregation. The monolayer chondrogenic culture with or without additionalβig-h3 was performed. Similar results were found via immunofluorescence staining of Sox 9 (Figure 5D) and western blots (Figure 5E). Thus, taken together, βig-h3 might induce chondrogenic differentiation of iTDSCs via promoting condensations and chondrogenic differentiation.

## DISCUSSION

Heterotopic ossification commonly happens at all sites of the body, and there are two broad categories: non-cell-mediated HO which is characterized by the direct deposition of calcium salts and cell-mediated HO which occurs when osteoblasts produce histologically normal bone. The cell-mediated HO can also be divided into two kinds, intramembranous ossification and endochondral ossification. Endochondral ossification refers to the process of embryonic formation of long bone as well as most of the acquired heterotopic ossification developed after trauma, burns, neurologic injuries, local inflammatory, or various surgeries, which begins with the differentiation and hypertrophy of chondrocytes, and then replaced by osteoblasts. The rarer genetic disorders like the fibrodysplasia ossificans progressive (FOP), which is caused by a gain-of-function mutation of the bone morphogenetic protein (BMP) type I receptor, is also formed via endochondral ossification [26]. The reason for the beginning of endochondral ossification remains unknown, mostly, it is considered as an error differentiation process of the progenitor cells recruited, in another word, the error differentiation into chondrocytes but not into the resident cell type for regeneration.

Regarding the special tendon ossification, it is interesting and important to notice the close relationship between the tendon and cartilage tissue, as well as the great many similarities they share. Will the similarities be the reason for the error differentiation into cartilage but not tenocytes? The tendon morphogenesis is strongly associated with chondrogenesis during embryonic development. Also, though Scleraxis (Scx) is typically considered as a tendon marker, it plays as an important regulator of gene expression in chondrogenesis [27]. And a unique pool of progenitor cells expressing both Sox9 and Scx has been characterized that may contribute to the establishment of the junction between cartilage and tendon [7, 28].

Tgfβ signaling has been proposed as key signals modulating connective tissue differentiation in embryonic and adult systems. Tgfβ modulates the balance between cartilage and tendon differentiation of limb mesodermal progenitors via the regulating Sox9 and Scx [8]. In vivo application of exogenous Tgfβs to the interdigital embryonic limb also induces ectopic cartilages, however, similar treatments in early limb mesenchyme exert an anti-chondrogenic influence [29]. In vitro, some studies have reported additional Tgfβ increases chondrogenesis of limb mesenchymal cells cultured at high density [30], as well as other cell lineages including bone marrow-derived chondrogenic stem cells [31]. However, other studies reported the chondrogenic differentiation effect of high-density mesenchymal cell cultures is reverted to fibrogenic when Tgfbs are added [32, 33]. The basis for the fibrous- versus chondrogenic- differentiation remains to be clarified, but some transcription factors downstream Tgfβ signaling such as Tgif 1 or SnoN [8], may serve a relevant function in this process. Tgfβs play critical roles in regulating chondrocyte differentiation from early to terminal stages, and mesenchymal condensation is no exception. In vitro studies demonstrate that Tgfβ1 induces mesenchymal cell condensation via the up-regulation of N-cadherin and FN [34, 35]. Tgfβ2 and Tgfβ3 are even more effective, causing a twofold greater accumulation of glycosaminoglycan [36]. Here, we also demonstrated the additional Tgfβ in iTDSCs cultures resulted in an accelerated chondrogenic differentiation effect, and βig-h3 may be an intermediate of it.

βig-h3 was first identified in human lung adenocarcinoma cells, known as an extracellular matrix protein that modulates cell adhesion, migration, growth, tumorigenesis, wound healing, and apoptosis. The protein is an ECM protein induced by Tgfβ and expressed in a wide variety of tissues, particular, it is strongly expressed in the perichondrium, periosteum, and pre-hypertrophic chondrocytes in articular cartilage and growth plate cartilage during endochondral ossification [17]. βig-h3 has a high affinity for other ECM proteins, including collagen, laminin, and fibronectin [39], thus facilitates cell-collagen interactions [19, 20, 38]. These findings suggest the interactions between βig-h3 and other ECM proteins may be an important part of the ECM homeostasis and chondrogenic differentiation discussed here. Transcripts of βig-h3 are very abundant around the tip of the growing digits and developing joints [15], indicating its role in promoting cell adhesion to establish the prechondrogenic aggregates of cartilage mesodermal progenitors [11]. Interestingly, βig-h3 is also remarkably expressed in the entheses of the tendon, the structural transit between cartilage and tendon [39], indicating its role of distinguishing the differentiation fate of progenitors into chondrocytes or tenocytes. In a series of researches based on cancer cell lines, βig-h3 is found to have a strong functional interaction with hypoxia-inducible factor-1 a (Hif-1a), a key pro-chondrogenic factor that directs the differentiation to chondrocytes in hypoxic microenvironment [40–42].

Taken together, the present study found βig-h3 was highly expressed in the inflammation and chondrogenesis stages of a heterotopic ossification model after rat Achilles tendon injury, as well as upon chondrogenic differentiation conditions in vitro. βig-h3 functioned as an ECM protein, which was induced by TGFβ signaling, could bind to the iTDSCs and inhibit the attachment of iTDSCs to collagen I. Exogenous βig-h3 can accelerate the process of mesenchymal condensation, promote chondrogenic differentiation, and finally promote endochondral ossification.

## MATERIALS AND METHODS

### Animals

All animal studies were conducted with approval by the Institutional Animal Care and Use Committee of the Affiliated Dongfang Hospital of Tongji University. The female Sprague-Dawley rats of 4-week-old were purchased from the Shanghai Slac Laboratory Animal Co.Ltd.

### Tendon surgery and cell transplantation

A complete transverse incision, without any attempt of repair, was made at the midpoint of the right Achilles tendon in 4-week-old female SD rats (Figure 1A). A total of 48 rats were euthanized 0, 1, 2, 4, 8 and 12 weeks post-operatively and tendon tissues were harvested to isolate progenitor cells or perform histological, immunohistochemical or gene expression analysis. At the same time, 12 additional rats were injected with/without rβig-h3 (300 μg/kg) at the areas near the tendon-to-bone attachment at 2 weeks post-surgery to figure out if rβig-h3 could enhance ossification directly. The rats were anesthetized 4 and 8 weeks post-surgery and subjected to X-ray analysis using a Philips digital radiography to confirm the formation of ectopic bone tissues.

### Isolation and culture of the tendon-derived stem cells (TDSCs)

Tendon-derived stem cells (TDSCs) were isolated from uninjured and injured tendons following the method previously reported [43]. Briefly, the tendon or fibrocartilage tissues that formed in the injured Achilles tendons (Figure 1B) were collected 1-week post-surgery. The tissues were minced and then incubated with 2.5 unit/ml Dispase (Mkbio, Shanghai) and 600U/ml type I collagenase (Mkbio, Shanghai) in PBS for 1h at 37°C with gentle shaking. The dissociated cells were harvested and plated on the 60-mm culture dishes at a density of about 100 cells/cm2 and cultured in DMEM containing 10% FBS (Sigma), 100U/mL penicillin (Sigma), 100 mg/mL streptomycin (Sigma), and 2mM L-glutamine (Sigma). The cells were cultured in the incubator of 37°C and 5% CO2, and passage 3 were used for all the cell experiments.

### Macrophage-conditioned media

Macrophages were isolated from rat peritoneal in this study. Briefly, 5 ml of aseptic paraffin wax was injected into the peritoneal of 3-week-old SD rats. The rats were euthanized 4 days later, soaked with 70% alcohol, and then make a small incision along the midline with sterile scissors to expose the intact peritoneal wall. Then 10 ml of PBS was injected into the peritoneal from the incision, shake the abdomen and then aspirate fluid from peritoneum using the same syringe and needle. Centrifuge the aspirated fluid in a refrigerated centrifuge of 4°C for 10 min at 400 × *g*. Then resuspend the cells at the bottom of the tube with DMEM/F12-10 (Gibco) and seeded the cells to Transwell® polyester membranes (Corning 3460) with the concentration of 1 × 10^6^ cells/well. The plates were incubated at 37 °C for 2 hours and the medium was changed to remove the unadhered cells.

### Co-culture of macrophage and TDSCs

iTDSCs were used to co-culture with macrophage cells. The iTDSCs were seeded on the bottom of 12-well plates at the density of 5 × 10^5^ cells per well. And the macrophages seeded on the Transwell® polyester membranes as described above. Both cells population were cultured in DMEM with 10% FBS, supplied with/without anti-βig-h3 antibody. And the iTDSCs were also cultured with the DMEM medium supplied with 10% FBS and TGF-β3 (10 ng/ml) or OriCell SD Rat Mesenchymal Stem Cell Chondrogenic Differentiation Basal Medium (Cyagen Biosciences) for control. The co-culture system was maintained for 72 h, and then the cell samples were collected for further analysis.

### Cell attachment analysis

For cell attachment assays, culture media was removed from the TDSCs/iTDSCs and then resuspended in DMEM containing 0.5% FBS before counting with a hemocytometer. 96-well plates were pre-coated with 10 μg/ml rat tail collagen type I (100 μl/well) (Mkbio, Shanghai), 10 μg/ml of bovine serum albumin (100 μl/well) (BSA, Beyotime), 10 μg/ml of fibronectin (100 μl/well) (FN, R&D), or 10 μg/ml of rβig-h3 (100 μl/well) (R&D) and incubated overnight at 4 °C. After removing excess liquid from the wells, the cells were immediately seeded in triplicate (2000 cells/well) to collagen I/BSA/FN/ rβig-h3 -coated plates and permitted to attach for 45 min at 37 °C (95% air containing 5% CO2) before gently rinsing off the unattached cells twice with PBS. Finally, toluidine blue was added to each well for 5 min at room temperature, rinsed three times and solubilized with 1% SDS (250 μl). The cell numbers were determined by reading absorbance at 595 nm using a plate reader (Biorad, Hercules, CA) and comparing to a standard curve.

Furthermore, the TDSCs/iTDSCs were resuspended in DMEM containing 0.5% FBS at a concentration of 2000 cells/200 μl containing 25 μg/ml of BSA, 25 μg/ml of FN, 25 μg/ml of rβig-h3, or nothing. For antibody blocking experiments, an equimolar amount of anti-βig-h3 antibody (R&D) was pre-incubated with rβig-h3 (25 μg/ml, R&D Systems, Minneapolis, MN) for 1 hr at room temperature before the addition of cells. Cells were immediately seeded in triplicate (2000 cells/well) to collagen I -coated plates and counted with toluidine blue with the method described above.

### Chondrogenic differentiation of iTDSCs

Both the micromass culture and monolayer culture methods were used to analyze the chondrogenic differentiation ability of the iTDSCs. The micromass culture method is performed as previously reported [44]. Briefly, the cells of a T75 flask (about 5*106) were collected and centrifugated at 1000rpm for 5 min. Aspirate the medium and resuspend the cells in the final volume (50ul per well*24 well). Add cells to the center of each well in a pre-coated (βig-h3, 25 μg/ml) 24-well plate and allow the cells to attach for 4 hours in the incubator. Then, add the OriCell SD Rat Mesenchymal Stem Cell Chondrogenic Differentiation Basal Medium carefully to each well without disturbing the cells. The monolayer culture is performed with common procedures, briefly, 5 × 105 cells were planted to each well of a 24-well plate and the Chondrogenic Differentiation Basal Medium is added when the cells reach 100% confluence. The culture medium is changed every 3 days and the cells were cultured for 7 days.

### Histological and immunohistochemical analyses

The Achilles Tendon was harvest and fixed with 4% (v/v) paraformaldehyde, decalcified with EDTA and then embedded in paraffin. Longitudinal sections of the Achilles tendons were made and subjected to histological staining with hematoxylin/eosin or Safranine O-Fast Green.

For the detection of TGFβ1, phospho-Smad2/3, βig-h3, CD68, and Nanog proteins, the sections were first deparaffinized and antigen retrieved with Tris-EDTA buffer. Then the sections were treated with PBST (0.25% Triton X-100) and blocked with 1% BSA. The primary antibodies were then incubated overnight at 4°C. Following washes with PBS, the sections were incubated with goat anti-rabbit biotinylated secondary antibody (1:200, Vector Laboratories) at room temperature for an hour, and incubated with ABC reagent (Vector Laboratories) for an hour followed by visualization of the antibody with ImmPACT DAB (Vector Laboratories) and counterstaining with hematoxylin.

### Immunofluorescence staining

For immunofluorescence staining, the cells were first fixed with 4% (v/v) paraformaldehyde for 15 mins, washed by PBST (0.25% Triton X-100) for 30 mins and blocked with 1% BSA for 30 mins. Then the cells were incubated with primary antibodies for 1 h at 37°C, followed by incubation with secondary fluorescence antibodies for 30 mins and finally covered with DAPI histology mounting medium.

### RNA isolation and gene expression analysis

Total RNA was isolated using trizol (Beyotime) from tissues and cells following the manufacturer’s protocol. The RNA was reverse-transcribed into cDNA using the reverse transcriptase (Takara, Japan). The cDNA was used for real-time polymerase chain reaction (Rt-PCR) with SYBR® Green (Takara, Japan) following the manufacturer’s protocol on the ABI Prism 7500 Fast System (Applied Biosystems, Carlsbad, CA, USA). The average threshold cycle value (Ct value) was calculated and normalized to that of the housekeeping gene GAPDH.

### Western blot

Total protein was obtained from the cells with RIPA lysis buffer (Beyotime) following the manufacturer’s protocol. The concentration of protein was measured using BCA Protein Assay Kit (Beyotime), and then equal amounts of protein samples were separated with sodium dodecyl sulfate-polyacrylamide gel electrophoresis and finally transferred onto nitrocellulose membranes (Beyotime). The membranes were then blocked with BSA for 30 mins and incubated with primary antibodies at 4 °C overnight, followed by the secondary antibody for 1 h. Immunoreactive protein bands were detected using an Odyssey scanning system (SYSTEM/Manufacturer Info). The protein expression levels of the kinases were normalized against β-actin.

### Statistical analyses

GraphPad Prism 7 was used for statistical analyses. All data were expressed as the mean value ± standard deviation (SD). Statistical significance was assessed by using a one-way analysis of variance (ANOVA). A probability value (p) of less than 0.05 was considered statistically significant.
